# Remarks on the Treatment of Phthisis by Drs. Condie, Riofrey, and Others, of Philadelphia

**Published:** 1850-03

**Authors:** 


*Remarks on the treatment of Phthisis by Drs. Condie, Riofrey, and others, of Philadelphia.*

The supposition that consumption is not a disease of warm climates, has been shown, by accurate statistics, to be unfounded. In the leading Southern cities of our own country, the disease is of very frequent occurrence. The natives of even the vaunted localities of Madeira, Italy, and the South of France, to which it was the common practice, not long ago, to send patients predisposed to consumption for a renewal of their health, have been found liable to the disease, if not in the same ratio as those of more northern and variable climates, at least to an extent sufficient to prove that warmth and comparative equability of temperature are no proteetives from its incursion and deadly march. Since more attention has been paid than was formerly done, to the collection of correct statistics, consumption has been shown to be the disease rather of civilized life, than of locality or climate. There can be no doubt that in a large number of cases, the predisposition to consumption is hereditary, still Dr. C. is convinced that the disease may be acquired by whatever has a tendency to lower gradually the energies of the living organism, and to impair hæmatosis and nutrition. Hence long continued sedentary occupations, in-door confinement, particularly in narrow ill-ventilated apartments, insufficient nutriment, close mental occupation, unbroken by relaxation and active exercise, anxiety of mind and the like, especially in the young, may produce consumption, where the constitution has inherited not the slightest tendency to the disease. It has been supposed to be the especial disease of the more enlightened and intelligent classes; this is likewise an error which has resulted from a neglect of accurate statistics. The poor and depraved of nearly all large cities are much more liable to consumption than the wealthy and intelligent; let the annals of our alms-houses and our prisons be consulted, and this will become apparent. In examining the bodies of the children of that portion of the population who inhabit the small, ill-ventilated houses of the narrow courts, lanes, and alleys of our city and its suburbs, Dr. C. has found, in the greater number of instances, whatever may have been the disease by which their death was occasioned, tubercles to exist in many of the organs, including the lungs. In regard to the treatment of consumption, Dr. C. had but little to say. He was convinced that no treatment would be successful, unless the patient were subjected to those hygienic influences which are calculated to improve the vital energies of the organism, to promote heaithy hæmatosis, and active regular nutrition of every tissue and organ of the body. That active daily exercise, in the open air, on foot or on horseback, plain, wholesome and sufficient food, and pleasurable excitement, confined within proper bounds, of the mind, has prevented the development of the disease in the predisposed, or even arrested its progress in its earlier stages, he has seen a sufficient number of facts to convince him. Some twenty-five years ago, Dr. C. was consulted by a young man laboring under the symptoms of incipient phthisis; tubercles had already been developed in the upper portion of the lungs. He was a journeyman saddler by trade, with a wife and family to support by his daily labor, carried often far into the night. He was candidly told by the Doctor that little or no benefit would be derived from any treatment, unless he could change his occupation for one which would enable him to take active daily exercise in the open air. This change he supposed, at the time, to be impossible for him to make, and after a short attendance, the Doctor discontinued his visits, and entirely lost sight of him, About ten years afterwards, the Doctor was accosted in the street by a person of a fine robust and healthful appearance, who made himself known as the very consumptive patient, whom ten years previously he had seen bending over his work, pale, emaciated, with a short, quick respiration, and a frequent, dry, harassing cough. The Doctor was surprised at the change which he had undergone, and inquired of him how that change had been effected. He replied, that after the Doctor had discontinued his visits, finding himself getting daily worse, and less able to work at his trade, he had seriously reflected upon what had been said in regard to a change of occupation, and following out the Doctor's advice, he had engaged himself to a vender of oysters, to haul them about the streets, with a horse and cart. Though much exposed to the weather, he soon found his health and strength to improve. While thus occupied, he was induced to go to a neighboring city to drive a dray for a merchant, by whom a capital was advanced to him to purchase a dray and horse for himself, and as he had few competitors in his business, he prospered in a pecuniary point of view, while his health at the same time, became fully re-established. Dr. C. did not adduce this case as an exemplification of the proper course to be pursued in every attack of consumption, but as an example of the powerful influence exerted over the early manifestations of the disease, by changing a sedentary, in-door occupation, requiring a constrained position of the body for many consecutive hours, for active exercise out of doors, even when attended with much exposure; an insufficient diet for one more abundant and nutritious, and, no doubt, also, cheerfulness and confidence of mind for the cheerless, depressing feelings, resulting from a consciousness that his constantly increasing debility would render him less and less able to minister to the wants of his family. In even an advanced stage of consumption, well regulated hygienic means were all important, but Dr. C. considered that it was only in the very commencement of the disease that they can be expected to exert an influence tending to the arrest of its development.

Dr. Riofrey, of Paris, who was present by invitation, was requested to give his views on the subject of Consumption.

Dr. Riofrey remarked, that he found the views of the medical men of Philadelphia, in relation to Consumption, more advanced, and more in accordance with what he considered sound practical experience, than those entertained by physicians elsewhere. In Paris there were still many practitioners who regard phthisis as an inflammatory disease, and keep their patients confined to their rooms on a low diet. Medicines are still in vogue, such as iodine, digitalis, &c., which can produce only a temporary effect upon the symptoms, without touching the source of the disease; they cannot improve the nutrition of the body, which is the grand object to be aimed at. In France, Dr. R.'s opinions on this disease were considered peculiar, but here he finds them prevalent, and amply confirmed by the experience of the leading members of the profession.

Dr. Riofrey referred to a series of papers on consumption, by the late Dr. Joseph Parrish, which were published in the year 1829–30, in the North American Medical and Surgical Journal. These papers he looked upon as presenting the most correct views in relation to the pathology and treatment of the disease in question.

Dr. Parrish, in these papers, discards the old idea of tubercles being the result of inflammation; he points out the intimate relation between scrofula and consumption, and the importance of favoring external scrofula, rather than dispersing it by local applications, and he shows that the true method of curing consumption, is by adopting measures to improve the nutrition; pure air, exercise, good diet, &c. He was induced to consider all these views as particularly sound. Taking Dr. Parrish's opinions in detail, Dr. R. might find some points upon which he could not agree, but the general tenor of the views, and the practice based upon them are highly instructive and valuable. The influence which these papers has had upon medical opinion in this country, was very evident, from all that Dr. R. had learned, since he had come to this city.

In his investigation into the curability of consumption, a subject which has occupied m uch of his attention of late years, Drr R. has found that all the recoveries from the disease have taken place under an invigorating plan, having for its object the improvement of the nutrition. It is upon this principle that the cod liver oil acts. It is an aliment easily assimilated, and gives to the constitution the elements which are wanting. Dr. Riofrey was among the first physicians to employ this article in consumption. He commenced the use in London, in 1835, from having heard of its good effects through Dr. Pearson of that city, who was using it in chronic rheumatism with great advantage. Its utility in scrofulous complaints, was first inferred, from the fact of the liver of the cod, being extensively employed as a domestic remedy in these affections in some parts of Germany. Mothers there give it to delicate children, without the advice of the physician, and find that they grow fat under its use. As the liver of the cod contains a large amount of oil, it was inferred that its remedial powers were attributable to it, and hence the oil was extracted and brought into general use.

Dr. R. was fearful that it might attain undue importance, to the exclusion of important hygienic remedies. It is no panacea, but an adjuvant to other means of improving the nutrition. The cases in which he had found it most useful, were those in which external scrofula and tubercles co-existed; but exercise, pure air, good and sufficient diet must be enjoined with it, in order to produce a beneficial effect. A good nutrition must be procured, before the tubercle can be overcome.

Dr. Samuel Jackson, (late of Northumberland,) thought Dr. Riofrey was right, in attributing the present advanced state of medical opinion on the nature and treatment of consumption in this city, especially to the influence of the late Dr. Parrish; his papers upon that subject, produced at the period of their publication a strong impression upon the minds of the profession, and an evident change in the views entertained of the nature of consumption, and the treatment pursued for its arrest and cure.

Dr. Pepper, believed that the true principle of treating phthisis, was by improving the nutrition, by means of pure air, out-door exercise, nutritious food, social enjoyments, &c. He knew cod liver oil had been useful in some cases, but he doubted whether it possessed any special virtue over other animal oils. A medical friend, in consequence of the frequent sophistication of the article, had substituted for it spermaceti oil, in the treatment of some cases of chronic rheumatism and scrofula, and apparently obtained from the substitute, the same good effects, as from the cod liver oil itself.

Dr. P. did not think that the iodine, in the oil of cod, had much to do with its virtues, nor did he suppose with some writers, that the constituents of bile which it contains were especially useful, yet he had certainly seen good effects from its use.

Dr. Emerson, wished to inquire of Dr. Riofrey, as to the prevalence of consumption in Paris; he supposed that the percentage of deaths from this disease in that city must be large, from the immense number of the population who were placed under the most unfavorable hygienic conditions.

He also wished to be informed of the state of opinion in Europe, in regard to the contagiousness of the disease.

Dr. Riofrey replied, that a fair estimate of the number of deaths from consumption in Paris, might be arrived at, from the fact, that nearly one-fourth of the deaths in the population of that city, occur in hospitals. These institutious are free to all, and from the admirable system upon which they are conducted, and the slight prejudice which exists against them, they are very much resorted to, not only by the poor, but by those in better circumstances. It is estimated that 1 in every 4.50 of the population of the hospitals die of consumption; this cannot be owing to the climate of Paris, for that is pure and temperate; there are other causes existing peculiar to that city; amongst these may be mentioned, the bad construction and crowded state of the houses, the sleeping apartments are very small, they get no air from chimneys, and have no other means of ventilation. This prevails universally among the rich and poor. The effect of alimentation, even when this is good, is impaired by the effects of a congned and impure domestic atmosphere, and all suffer alike from this cause, from the porter to the lady of rank.

In regard to the belief in the contagiousness of consumption, this prevails very generally among the people in the provinces of France, and very generally also in Spain. At Montpellier, he found it to be firmly believed by the community; but by the intelligent physicians of Europe, the idea of the contagiousness of consumption was generally repudiated.

Dr. Riofrey remarked at length on the subject of climative influence, in the production of consumption. He believed this branch of science had not been investigated, in a manner calculated to lead to any precise results, and that our knowledge in regard to it, was, as yet, very unsatisfactory. There have been few treatises on the subject of climate, published in America, none he believed excepting that of Dr. Forry, of New York.

In Europe, even, but few works have been written on this subject. That of Sir James Clark, is, perhaps, the most elaborate and authoritative; it is, nevertheless. full of errors. Many of the assertions in this work have been proved by recent statistical results to be inaccurate.

Dr. Cary has recently published a work which is more correct; and which contradicts many of the statements made by Dr. Clark. Dr. C. has been investigating the climate of Italy for ten years, and has found that the cities of Italy, in which consumption prevails to the least extent, were Venice and Milan.

Dr. Riofrey deprecated the usual practice of sending patients away from their homes, when far advanced in consumption, from a fancied idea of benefit of change of climate. He believed many of them only went away to die.

Dr. Hays considered the chief advantage of sending patients to the South during the winter, was that they might be enabled to enjoy out-door exercise; our northern climate is too changeable, cold, and inclement to allow of this, excepting during the summer, and early portion of the autumn.

Dr. Riofrey believed the opinion which referred the production of tubercules to a defective nutrition, to be correct. He was persuaded that they would be much more prevalent in this country than they are, if the people generally had not the means of obtaining a full supply of good food. It was an alimentation and not simply, he repeated, as a medicine, that he recommended the oil of cod in consumption; he did not believe it was the small portion of bromine or iodine contained in it, which rendered it useful, but rather its capacity of being rapidly assimilated and of making fat. He had found some patients who could not take this article, they seemed to have a natural antipathy to it, and in others it produced sickness and vomiting. In these cases its employment should not be insisted on. In some children who take it readily, he has found it to have a wonderful effect. After using it a few months, he has known attenuated delicate children to become fat and robust. It is an excellent alimentation in these cases, and as such alone it should be regarded.—*Transactions of the College of Physicians of Philadelphia.*

